# Draft Genome Assembly of *Ralstonia pickettii* Type Strain K-288 (ATCC 27853)

**DOI:** 10.1128/genomeA.00973-14

**Published:** 2014-09-25

**Authors:** H. E. Daligault, K. W. Davenport, T. D. Minogue, S. M. Broomall, D. C. Bruce, P. S. Chain, S. R. Coyne, H. S. Gibbons, J. Jaissle, C.-C. Lo, L. Meincke, A. C. Munk, C. N. Rosenzweig, S. L. Johnson

**Affiliations:** a Diagnostic Systems Division (DSD), United States Army Medical Research Institute of Infectious Diseases (USAMRIID), Fort Detrick, Maryland, USA; b Los Alamos National Laboratory, Los Alamos, New Mexico, USA; c United States Army Edgewood Chemical Biological Center (ECBC), Aberdeen Proving Ground, Aberdeen, Maryland, USA

## Abstract

We present the genome assembly of *Ralstonia pickettii* K-288 (ATCC 27511), consisting of 27 contigs placed into a single scaffold. This 4.76-Mbp genome has 64.0% G+C content and 4,425 coding sequences. Because this is the type strain, inclusion of its data set among other *Ralstonia* genomes should provide a historical genomic perspective.

## GENOME ANNOUNCEMENT

*Ralstonia pickettii* is a Gram-negative rod-shaped member of the β-proteobacteria commonly found in moist environments and is an increasing cause of nosocomial human infection (1, 2). *R. pickettii* K-288 (ATCC 27511) is the type strain of the genus, originally isolated from a tracheotomy patient. As of this writing, only six genome assemblies of *R. pickettii* (3 complete and 3 draft) are available in the public database and none for this type strain, which was originally described in 1973 (3).

High-quality genomic DNA was extracted from a 100-mL bacterial culture of a purified isolate using the QIAgen Genomic Tip-500 at the USAMRIID Diagnostic Systems Division (DSD). Draft sequence data included a 100-bp Illumina library (279-fold genome coverage) and a separate long-insert paired-end library (9,590- ± 2,397-bp insert, 42-fold genome coverage) (Roche 454 Titanium platform). The two data sets were assembled together in Newbler (Roche, version 2.6) and the consensus sequences were computationally shredded into 2-kbp overlapping fake reads (shreds). The raw reads were also assembled in Velvet (version 1.1.05), and those consensus sequences were computationally shredded into 1.5-kbp overlapping shreds (4). All draft data were then assembled together in Allpaths (version 39750), and the consensus sequences were computationally shredded into 10-kbp overlapping shreds (5). We then integrated the Newbler consensus shreds, Velvet consensus shreds, Allpaths consensus shreds, and a subset of the long-insert read pairs using parallel Phrap version SPS-4.24 (High Performance Software, LLC). Possible misassemblies were corrected, and some gap closure was accomplished with manual editing in Consed (6–8).

Automatic annotation of the *R. pickettii* K-288 genome utilized an Ergatis-based workflow at LANL with minor manual curation. The final annotated assembly includes 4,425 coding sequences, 5 rRNAs, and 49 tRNAs in the 4,762,999-bp genome.

### Nucleotide sequence accession number.

This genome is available in NCBI under the accession number JOVL00000000.
